# Mammographic texture and risk of breast cancer by tumor type and estrogen receptor status

**DOI:** 10.1186/s13058-016-0778-1

**Published:** 2016-12-06

**Authors:** Serghei Malkov, John A. Shepherd, Christopher G. Scott, Rulla M. Tamimi, Lin Ma, Kimberly A. Bertrand, Fergus Couch, Matthew R. Jensen, Amir P. Mahmoudzadeh, Bo Fan, Aaron Norman, Kathleen R. Brandt, V. Shane Pankratz, Celine M. Vachon, Karla Kerlikowske

**Affiliations:** 1 Department of Radiology and Biomedical Imaging, UCSF School of Medicine, San Francisco, CA USA; 2 Mayo Clinic, Rochester, MN USA; 3 UCSF Departments of Medicine and Epidemiology/Biostatistics, San Francisco, CA USA; 4 Harvard Medical School, Boston, MA USA; 5 Slone Epidemiology Center at Boston University, Boston, MA USA

## Abstract

**Background:**

Several studies have shown that mammographic texture features are associated with breast cancer risk independent of the contribution of breast density. Thus, texture features may provide novel information for risk stratification. We examined the association of a set of established texture features with breast cancer risk by tumor type and estrogen receptor (ER) status, accounting for breast density.

**Methods:**

This study combines five case–control studies including 1171 breast cancer cases and 1659 controls matched for age, date of mammogram, and study. Mammographic breast density and 46 breast texture features, including first- and second-order features, Fourier transform, and fractal dimension analysis, were evaluated from digitized film-screen mammograms. Logistic regression models evaluated each normalized feature with breast cancer after adjustment for age, body mass index, first-degree family history, percent density, and study.

**Results:**

Of the mammographic features analyzed, fractal dimension and second-order statistics features were significantly associated (*p* < 0.05) with breast cancer. Fractal dimensions for the thresholds equal to 10% and 15% (*FD_TH10* and *FD_TH15)* were associated with an increased risk of breast cancer while thresholds from 60% to 85% (*FD_TH60* to *FD_TH85)* were associated with a decreased risk. Increasing the *FD_TH75* and *Energy* feature values were associated with a decreased risk of breast cancer while increasing *Entropy* was associated with a decreased risk of breast cancer. For example, 1 standard deviation increase of FD_TH75 was associated with a 13% reduced risk of breast cancer (odds ratio = 0.87, 95% confidence interval 0.79–0.95). Overall, the direction of associations between features and ductal carcinoma in situ (DCIS) and invasive cancer, and estrogen receptor positive and negative cancer were similar.

**Conclusion:**

Mammographic features derived from film-screen mammograms are associated with breast cancer risk independent of percent mammographic density. Some texture features also demonstrated associations for specific tumor types. For future work, we plan to assess risk prediction combining mammographic density and features assessed on digital images.

**Electronic supplementary material:**

The online version of this article (doi:10.1186/s13058-016-0778-1) contains supplementary material, which is available to authorized users.

## Background

Women with mammographically dense breasts are at a higher risk of developing breast cancer than women with more fatty breasts. The risk of developing breast cancer can be four- to six-times higher in women with breast density in the top quartile of the population compared to the bottom quartile [1, 2]. Why breast density is predictive of future cancer occurrence is not fully known. What is known is that breast density is not homogeneous. Some of the earliest measures of breast density categorized the appearance of mammograms by the patterns projected from the heterogeneity of the tissue [3]. However, the description of the heterogeneity, or “texture”, has not been incorporated in standardization reporting of breast density categories in the Breast Imaging-Reporting and Data System (BI-RADS) [4], or the quantitative measures of volumetric breast density using methods such as the Volpara (Matakina, Wellington, New Zealand) and Quantra (Hologic, Inc., Marlborough, MA, USA) [5].

Breast density texture can be described using numerous statistical descriptors of the distribution and spatial relationship of grayscale values in the image pixels. Texture has been studied as a breast cancer risk factor independent of average breast density [6–11], but the results have not been adequately adjusted for breast density and other risk factors. For example, Byng et al. reported a negative significant correlation between regional skewness, fractal dimension, and cancer risk [7]. However, Torres-Mejia et al. [6] reported that the regional skewness and fractal dimensions had no association with breast cancer after adjusting for other risk factors and overall breast density. One feature, lacunarity, remained significant [6]. Manduca et al. found that skewness and kurtosis did not predict breast cancer risk [8], but did find associations for the Markovian, run length, Laws, wavelet, and Fourier transformations. After adjustment for planar mammographic percent density (PD), each feature attenuated only slightly and retained statistical significance; however, simultaneous inclusion of these features in a model with PD did not significantly improve the ability to predict breast cancer [8]. Other studies have shown that differences in texture and density features are related to predisposing mutations and tumor type including BRCA1/BRCA2 mutation carriers [12–14] and estrogen receptor (ER) status [15–17]. Thus, the density patterns of the parenchymal tissue have attracted clinical attention because of their potential to offer additional information about subtype and cancer biology. However, it remains unknown if breast texture descriptors will help better identify women at high risk of breast cancer from standard screening mammograms.

To this end, we amassed a library of imaging features previously reported on in the breast imaging and general imaging literature as candidate descriptors of breast tissue characteristics. In this study, we investigated the association of these descriptors and breast cancer risk using prospectively acquired mammograms from five breast cancer epidemiology studies. We also examined the association of these descriptors to tumor type and ER status.

## Methods

### Study design

This study is a large, comprehensive pooled analysis of five case–control studies, two of which were nested within cohorts, to examine the association between texture of mammographic density and breast cancer risk and breast cancer subtypes.

### Study population

The studies and populations used in this analysis have been previously described elsewhere [16]. Briefly, the participating studies included the Mayo Mammography Health Study (MMHS) [18], the Nurses’ Health Studies (NHS and NHSII) [19], the Mayo Clinic Mammography Study (MCMAM) [20], and the San Francisco Bay Area Breast Cancer SPORE and San Francisco Mammography Registry (SFMR) at the University of California San Francisco (UCSF) [21]. Breast cancer cases diagnosed within 6 months of mammography were excluded from all studies. We collected covariate data from medical record review (MCMAM), and self-administered questionnaires (NHS, NHSII, SFMR), or both (MMHS). Information was obtained before (NHS, NHSII) or at the time of (MMHS, MCMAM, SFMR) screening mammogram. The Institutional Review Boards at the Mayo Clinic, Brigham and Women’s Hospital, UCSF, and the Connecticut Department of Public Health Human Investigations Committee reviewed and approved these studies. Informed consent was obtained or implied by return of questionnaires (NHS, NHSII).

There were 9353 women with screening visits during the study period from all studies. For MMHS and SFMR only, due to study design, large batches of cases were digitized at one time followed later by batches of matched controls. Thus, to ensure no bias due to potential confounding by digitization we only included those cases and matched controls that were digitized in the same batches, resulting in a substantially reduced sample for these two studies. To ensure that no bias was associated with study exclusions due to digitizer in these two studies, we compared the included cancer cases to the excluded cancer cases. We found that the eligible vs. excluded cases did not differ in terms of their demographic and clinical characteristics (*P* > 0.05). Similarly, matched controls were compared against the whole study population and were found to be comparable (data not shown). Overall, 2830 women were eligible for our case–control set and 6523 (69.7% of population) from MMHS and SFMR were excluded. Of these, mammograms of 1171 breast cancer cases and 1659 controls were analyzed.

### Mammogram digitization and harmonization

For this study, the craniocaudal (cc) views of screening examinations of both breasts were digitized at each respective study site. The cc view images were more conducive to being analyzed automatically with our algorithms; also, not all studies had mediolateral oblique views available. The MMHS screen-film mammograms were digitized on the Array 2905 laser digitizer (Array Corporation, The Netherlands) that has 50-μm (limiting) pixel spacing with 12-bit grayscale bit depth. The MCMAM mammograms were digitized on a Lumiscan 85 scanner with 12-bit grayscale bit depth and 0.100 × 0.100 mm^2^ pixel size. For mammograms provided by the SFMR, digitization was performed using two digitizers, a R2 ImageChecker with 16-bit dynamic range and 150-μm pixel size, and a Vidar Diagnostic Pro (Vidar Systems Corporation) with 16-bit dynamic range and 169-μm pixel size. For NHS and NHSII, film mammograms were digitized at 261 μm per pixel with a Lumisys 85 laser film scanner (Lumisys, Sunnyvale, CA, USA) or a VIDAR CAD PRO Advantage scanner (VIDAR Systems Corporation, Herndon, VA, USA) and comparable resolution of 150 dots per inch and 12 bit depth. To minimize effects of the film digitization process, we performed a harmonization procedure by rescaling all images to have the same pixel size and dynamic range. The ultimate space resolution was set to 160 μm using a Matlab “imresize” function with default parameters (bicubic interpolation). The dynamic scale of all images was converted into 16-bit grayscale by the proper coefficient multiplication.

### Assessment of mammographic density

To quantify PD, two semi-automatic threshold techniques were applied: Cumulus [22] (all studies besides SFMR) and UCSF custom software [23] (SFMR study; comparable to Cumulus). The test at the beginning of the study demonstrated that there was high correlation between the UCSF and Cumulus methods. As documented in [16], similar results are obtained from an average of both breasts and from a randomly selected side. We quantified PD on the contralateral breast for cases and the corresponding side for matched controls for all studies except NHS and NHSII where the average PD of both left and right views were used. Only one reader read the images at each site. To match PD measures between readers and studies, we standardized the readings by removing the study-specific age trends, standardizing the variability across studies, and incorporating the known age trend in PD into the standardized PD. Details of this standardization procedure have been previously published [16].

### Breast texture measurements

We automated 46 candidate image texture features into our mammography image analysis program (Table 1). Features were measured on both left and right cc views for all subjects. The texture analysis was performed in the entire breast area. The entire breast area was automatically segmented from the background by global thresholding. Texture measures were grouped by the type of statistical description. Features derived from the histogram of the mammographic grayscale values were grouped as “Gray-Level Histogram” and include the image *Standard Deviation*, *Skewness*, *Kurtosis*, and *Balance* [7, 22, 24–26]. The second-order features described the spatial relationships between pixel intensities. We derived these second-order features using two matrixes: gray-level co-occurrence matrix (GLCM) [24, 25, 27] and neighborhood gray-tone difference matrix (NGTDM) [24, 28]. The GLCM matrix defined the distribution of co-occurring values at a given pixel offset in the image. Because co-occurrence matrices were often large and sparse, various metrics were used to describe the features of the matrix. The GLCM matrix was created by Matlab “graycomatrix” function with a number of gray levels equal to 16 and offset = [0 1] related to horizontal proximity of the pixels. The features used to describe a GLCM are often called Haralick features [27], and include *Energy*, *Entropy*, *Dissimilarity*, *Contrast*, *Homogeneity*, *Correlation*, *Mean* and *Variance*. In the textural analysis, the GLCM *Entropy* represents image pixel spatial disorder (e.g., heavy heterogeneous textures versus a flat gray level and smooth textures). The GLCM *Energy* represents local homogeneity and is a measure opposite to GLCM *Entropy*. Actually, this texture feature describes the degree of texture uniformity; basically, more homogeneous texture has a higher *Energy*. For example, the image with only constant grayscale pixels has *Energy* equal to 0. Other similar texture features from this table are GLCM *Homogeneity* and *Dissimilarity. Homogeneity* measures how uniform are the non-zero entries in the GLCM matrix. This feature represents existence of repetitions in texture. The image with irregular texture elements and their spatial positions is characterized by low *Homogeneity*. An image that contains repetitive structures represents high *Homogeneity. Dissimilarity* is a measure that defines the variation of gray level pairs in an image. It is very similar to *Contrast* with a difference in the weight.

**Table 1:** Image texture features that are currently defined for all study participants

Analysis groups	Texture features	Texture feature name	References
Gray-level histogram	Standard deviation	STD	[7, 22, 24–26]
	Skewness	Skewness	
	Kurtosis	Kurtosis	
	Balance	Balance	
Gray-level co-occurrence matrix (GLCM)	GLCM Energy	Energy	[24, 25, 27, 29]
	GLCM Entropy	Entropy	
	GLCM Dissimilarity	Dissimilarity	
	GLCM Contrast	Contrast	
	GLCM Homogeneity	Homogeneity	
	GLCM Correlation	Correlation	
	GLCM Mean	GLCM Mean	
	GLCM Variance	GLCM Variance	
Neighborhood gray-tone difference matrix (NGTDM)	NGTDM Coarseness	NGTDM Coarseness	[24, 28, 29]
	NGTDM Contrast	NGTDM Contrast	
	NGTDM Complexity	Complexity	
	NGTDM Strength	Strength	
	NGTDM Busyness	Busyness	
Edge frequency analysis	Mean gradient	Mean_Gradient	[29]
Fourier transform (FT) analysis, power spectrum	RMS (root mean square)	FT_RMS	[29]
	FMP (first moment of power spectrum)	FT_FMP	
	SMP (second moment of power spectrum)	FT_SMP	
	FD (fractal dimension) from power spectrum exponent	FT_FD	
Fractal analysis	Intercept of the plot of the standard deviation of the high frequency image as a function of the size the kernel	CD_Yint	[29–31]
	Continuous dimension (CD), slope and intercept	CD_Slope	
	HZ_PROJ	HZ_PROJ	
	FD of the standard deviation	FD_Sigma	
	FD of image using thresholds from 5%-85%	FD_TH_5: FD_TH_85	
	FD of the surface of the breast considering the gray value representing the height	FD_CALDWELL	
	FD, Minkowski method	FD_Minkowski	

The NGTDM is a column matrix, which was first defined by Amadasun and King [28]. This matrix was derived by calculating the gray level difference between pixels with a certain gray level and their neighboring pixels. The NGTDM features included were *Coarseness*, *Contrast*, *Complexity*, *Strength* and *Busyness* [24, 28]. One feature, the mean gradient, was from a group of features called the Edge Frequency Analysis group. Lastly, Fourier and fractal analysis groups defined the remainder of the features. Fourier transform (FT) operations were used to estimate features in the frequency domain: root mean square (*FT_RMS*), first (*FT_FMP*) and second (*FT_SMP*) moments of power spectrum, and fractal dimension (FD) from power spectrum exponent (*FT_FD*) [29]. To define fractal qualities, shapes within the image were created using the pixels at a percentage threshold value of the total contrast (i.e., *FD_TH_X*, for threshold at X = 5, 10, 15…85%). These features were derived by a box counting method. Further fractal features include FD of the standard deviation (*FD_Sigma*), intercept of the plot of the standard deviation of the high frequency image as a function of the size the kernel (*CD_Yint*), slope of the plot of the standard deviation of the high-frequency image as a function of the size the kernel (*CD_Slope*), standard deviation of the mean value of the breast pixels rows (*HZ_PROJ*), FD of the surface of the breast considering the gray value represents the height (*FD_CALDWELL*) [30, 31], and Minkowski fractal dimension (*FD_Minkowski*) derived from morphological image operations [29]. The *FD_Minkowski* is similar to the box counting fractal dimensions (i.e., *FD_TH* variables). It is calculated by an image dilation procedure with different scale structure disk element. As a result of edge frequency analysis, the mean gradient parameter was created. We previously demonstrated the utility of this set of features for derivation of volumetric breast density by a statistical model approach [32].

### Assessment of tumor characteristics

Tumor type (invasive vs. ductal carcinoma in situ (DCIS)) and ER status were available using Northern and Southern California Surveillance Epidemiology and End Results programs for SFMR, pathology reports or immunohistochemical analysis of tumor microarrays for NHS and NHSII, and state and clinic cancer registries for MMHS and MCMAM.

### Statistical analysis

Risk factors and PD phenotypes were harmonized on the eligible cases and controls. For all subjects, concordance between features measured on left and right sides were evaluated. Lin’s concordance correlation coefficients were used to summarize the correlation between left and right sides. Values ranged from 0.50 to 0.98 with median of 0.85. Given this, we chose to average sides to reduce noise in the measurements. To avoid issues with outliers and violations of distributional assumptions, the averaged features were normalized within each study using a normal transformation of the ranks. All analyses were performed using the normalized features. Logistic regression models evaluated the overall breast cancer associations with each normalized feature as a continuous variable and results are presented as odds ratio (OR) per 1 standard deviation (SD). All models were adjusted for age (continuous), body mass index (BMI) (continuous), first-degree family history of breast cancer (yes vs. no vs. unknown), PD (continuous), and study. To assess whether there were differences in associations by study, we included and tested an interaction term for texture feature by study. Study-specific results were also examined and summarized. The top 15 of 46 analyzed features that were significant (*p* < 0.05) in the case–control models were selected for further analysis. Polytomous logistic regression models were fitted to examine associations of features with respect to invasive/DCIS breast cancers and ER status. Contrasts were constructed within the polytomous model framework to test for differences of feature associations between tumor subgroups (p-het). SAS version 9.3 was used for analyses and two-sided *p* values < 0.05 were considered to be statistically significant. Pearson correlation coefficients were used to examine correlations among features and also correlations of features with PD among control subjects. Dendrograms were created to illustrate clustering among the significant features, age, body mass index (BMI), and PD on data from controls. A hierarchical clustering method using averaged distance was utilized as implemented in “proc cluster” in SAS.

## Results

The baseline case and control characteristics of the eligible population are shown in Table 2. The cases had stronger family history and were more likely to have higher PD compared with controls. Both cases and control groups were of similar age, BMI, menopause status, and parity. The baseline characteristics of the study population separated by study site are presented in Additional file 1 (Table S1). The NHSII site population is different from other sites by lower age, premenopausal prevalence, and higher PD. The baseline characteristics of study population separated by study site demonstrate similar trends between cancers and controls as above mentioned.

**Table 2:** Baseline characteristics of study population matched by age, date of mammogram, and study

	Cases	Controls
*N*	1171	1659
Mean age at mammogram (years)	55.4 (10.6)	55.3 (10.6)
Mean age at diagnosis (years)	60.5 (10.8)	–
Mean BMI (kg/m^2^)	25.7 (6.1)	25.8 (6.8)
Body mass index categories (kg/m^2^)*		
< 25	551 (47.1%)	854 (51.5%)
25–29	369 (31.5%)	443 (26.7%)
30–34	155 (13.2%)	196 (11.8%)
35+	73 (6.2%)	136 (8.2%)
Unknown	23 (2.0%)	30 (1.8%)
Menopausal Status		
Premenopausal	430 (36.7%)	632 (38.1%)
Postmenopausal	697 (59.5%)	962 (58%)
Unknown	44 (3.8%)	65 (3.9%)
Parity		
Nulliparous	169 (14.4%)	218 (13.1%)
Parous	977 (83.4%)	1415 (85.3%)
Unknown	25 (2.1%)	26 (1.6%)
Postmenopausal hormone therapy^a^*		
Not current	255 (51.5%)	367 (57.5%)
Current, estrogen	116 (23.4%)	156 (24.5%)
Current, estrogen + progestin	124 (25.1%)	115 (18.0%)
Family history*		
No	973 (83.1%)	1453 (87.6%)
Yes	196 (16.7%)	206 (12.4%)
Unknown	2 (0.2%)	0 (0%)
Standardized mean percent mammographic density (%)*	32.9 (18.7)	27.9 (18.4)
Standardized mean dense area (cm^2^)*	63.5 (43.1)	52.2 (37.8)
Standardized mean non-dense area (cm^2^)*	149.4 (98.7)	158.9 (102.1)

Data are presented as the mean (standard deviation) or number (%).

^a^Among postmenopausal women in MMHS, NHS, NHSII, and SFMR

*p < 0.05, cases versus controls

*BMI* body mass index

The top 15 of 46 analyzed features had a statistically significant (*p* < 0.05) association with breast cancer after adjustment for age, BMI, family history, PD, and study (Table 3). It should be noted that the features mostly follow the same trend across studies even though some are not significant in their separate OR estimation, and there was no evidence of study heterogeneity for any feature (*p* > 0.05 for all). Study-specific estimates for SFMR were often not consistent with other studies. In sensitivity analysis, we excluded SFMR to explore the impact of these differences and found similar results (data not shown). Three features with the strongest association were *FD_TH_75*, *Energy*, and *Entropy*. Increasing the *FD_TH_75* and *Energy* feature values were associated with a decreased risk of breast cancer while increasing *Entropy* was associated with an increased risk of breast cancer. The fractal dimension features were separated into two groups. The first group described the fractal dimensions in the densest pixels, and contained features *FD_TH_60*, *FD_TH_65*, *FD_TH_70*, *FD_TH_75*, *FD_TH_80*, *FD_TH_85*, and *FD_Minkowski*. All these features were significant and were associated with a decrease in cancer risk with the most significant association OR (95% confidence interval (CI)) per 1 SD = 0.87 (0.79–0.95) for *FD_TH_75*. The second feature group described fractal dimensions in the lower density (less opaque) pixels: *FD_TH_10* and *FD_TH_15*. In contrast to the first group, they were associated with an increase in breast cancer risk. *Energy* and *Entropy* demonstrate opposite associations to cancer with OR (95% CI) 0.88 (0.81–0.96) and 1.14 (1.05–1.25), respectively. The GLCM features *Homogeneity* and *Dissimilarity* showed opposite trends with OR (95% CI) 1.10 (1.01–1.20) and 0.91 (0.83–0.99), respectively. Table 3 also demonstrates the results of area under the curve (AUC) analysis of different feature models. For the baseline model (adjusted for age, BMI, family history, PD, and study), AUC was 0.617 and with with top feature (*FD_TH_75*) it was 0.621, suggesting modest increases in discrimination with the addition of this texture feature.

**Table 3:** The top 15 of 46 analyzed features were significant (*p* < 0.05) in the case–control models

Feature	All five studies OR (95% CI)	*p* value	AUC^a^	MMHS OR (95% CI)	MCMAM OR (95% CI)	SFMR OR (95% CI)	NHS OR (95% CI)	NHSII OR (95% CI)
*N* case/*N* control	1171/1659			62/112	242/395	104/206	412/454	351/492
*FD_TH_75*	*0.87 (0.79–0.95)*	*0.003*	0.621	*0.76 (0.48–1.20)*	0.73 (0.56–0.94)*	*1.28 (0.90–1.82)*	*0.94 (0.80–1.11)*	0.84 (0.72–0.98)*
*Energy*	0.88 (0.81–0.96)	0.003	0.621	1.03 (0.71–1.51)	0.84 (0.70–1.01)	1.35 (0.97–1.89)	0.85 (0.73–0.98)*	0.86 (0.73–1.00)
*Entropy*	*1.14 (1.05–1.25)*	*0.003*	0.620	*1.01 (0.68–1.51)*	1.30 (1.06–1.59)*	*0.75 (0.54–1.04)*	1.19 (1.02–1.38)*	*1.13 (0.96–1.32)*
*FD_TH_70*	*0.87 (0.79–0.96)*	*0.005*	0.621	*0.88 (0.55–1.43)*	0.72 (0.55–0.94)*	*1.28 (0.87–1.87)*	*0.97 (0.82–1.15)*	0.82 (0.70–0.96)*
*FD_TH_80*	0.89 (0.82–0.98)	0.012	0.620	0.86 (0.56–1.31)	0.88 (0.70–1.12)	1.17 (0.84–1.64)	0.90 (0.77–1.06)	0.87 (0.75–1.01)
*FD_TH_10*	1.11 (1.02–1.20)	0.015	0.620	1.43 (1.00–2.06)	1.11 (0.92–1.34)	1.07 (0.80–1.42)	1.03 (0.89–1.19)	1.15 (0.98–1.34)
*Kurtosis*	0.89 (0.81–0.98)	0.015	0.620	0.91 (0.61–1.34)	0.85 (0.69–1.06)	0.84 (0.60–1.16)	0.86 (0.74–1.01)	0.95 (0.80–1.12)
*FD_TH_65*	0.88 (0.80–0.98)	0.016	0.620	0.95 (0.57–1.58)	0.78 (0.59–1.04)	1.50 (0.98–2.29)	0.97 (0.82–1.15)	0.81 (0.68–0.96)*
*FD_Minkowski*	0.88 (0.79–0.98)	0.017	0.621	0.65 (0.34–1.24)	0.85 (0.67–1.09)	1.07 (0.72–1.59)	0.84 (0.70–1.00)*	0.91 (0.74–1.13)
*Busyness*	1.10 (1.02–1.20)	0.019	0.619	1.43 (0.97–2.13)	1.17 (0.97–1.41)	1.00 (0.74–1.35)	0.99 (0.86–1.15)	1.18 (1.02–1.38)*
*Homogeneity*	1.10 (1.01–1.20)	0.023	0.620	1.43 (0.95–2.14)	0.92 (0.76–1.13)	1.38 (1.00–1.90)	1.09 (0.94–1.26)	1.18 (1.01–1.37)*
*Dissimilarity*	0.91 (0.83–0.99)	0.033	0.620	0.72 (0.48–1.09)	1.10 (0.90–1.34)	0.74 (0.53–1.03)	0.92 (0.79–1.06)	0.85 (0.73–0.99)*
*FD_TH_60*	*0.89 (0.80–0.99)*	*0.035*	0.620	*1.02 (0.60–1.73)*	0.74 (0.55–0.98)*	*1.49 (0.95–2.33)*	*1.00 (0.83–1.19)*	0.82 (0.68–0.98)*
*FD_TH_85*	0.92 (0.84–1.00)	0.046	0.619	0.98 (0.65–1.46)	0.89 (0.71–1.11)	1.12 (0.82–1.53)	0.93 (0.79–1.08)	0.90 (0.78–1.05)
*FD_TH_15*	1.09 (1.00–1.18)	0.048	0.619	1.42 (0.96–2.11)	1.09 (0.91–1.31)	1.04 (0.79–1.39)	0.96 (0.83–1.11)	1.21 (1.04–1.42)*

Features listed in *italics* were significant in at least two studies.

Results are presented as odds ratio (OR) and 95% confidence interval (CI) per 1 standard deviation in normalized feature after adjustment for age, body mass index (BMI), family history, percent density (PD), and study.

^a^Adjusted for age, BMI, family history, PD, and study. Area under the curve (AUC) for the adjustment factors only is 0.617 (95% CI 0.596–0.638)

*Study *p* values < 0.05.

**Fig. 1:**
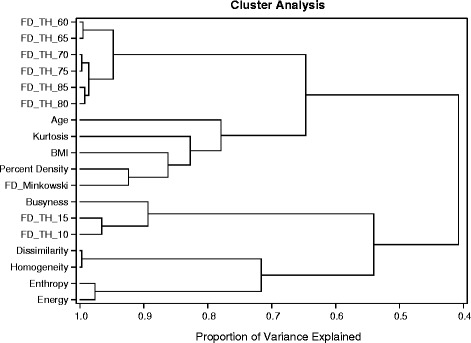
Dendrogram of cluster analysis of the top 15 features with PD, age, and BMI. Similar features cluster together. Percent density groups closely with body mass index (*BMI*) and age. The figure is restricted to the controls

Figure 1 shows the dendrogram noting the clustering of the top 15 features and clinical risk factors (PD, age, BMI) restricted to the control subjects (see Additional file 2: Figure S1 for clustering results restricted to the cases). The features separated into two primary clusters. Within the first cluster, features *FD_TH_60* through *FD_TH_85* formed a subcluster separate from the other non-feature risk factors. Interestingly, the clinical risk factors (BMI, age, PD) form a subcluster with *Kurtosis* and *Busyness* independent of other features. The second main cluster includes pairs of *Entropy/Energy*, *Dissimilarity*/*Homogeneity*, and *FD_TH_10*/*FD_TH_15*. The intercorrelation of each feature and risk factor calculated using control subjects is shown in Table 4 (see Additional file 1: Table S2 for intercorrelation calculated using case subjects). Interestingly PD is highly correlated to features similar to *FD_TH_75, FD_Minkowski* and *Kurtosis* from the same primary cluster group. However, the features of the second primary cluster show no or negligible association with PD.

**Table 4:**
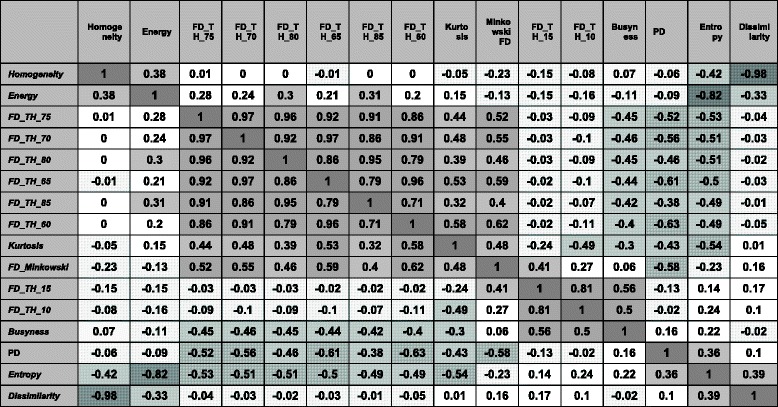
Pearson correlation coefficient for the top 15 significant features

Correlations calculated using control subjects. Gray and gray with line patterns highlight the strength of positive and negative associations, respectively

**Fig. 2:**
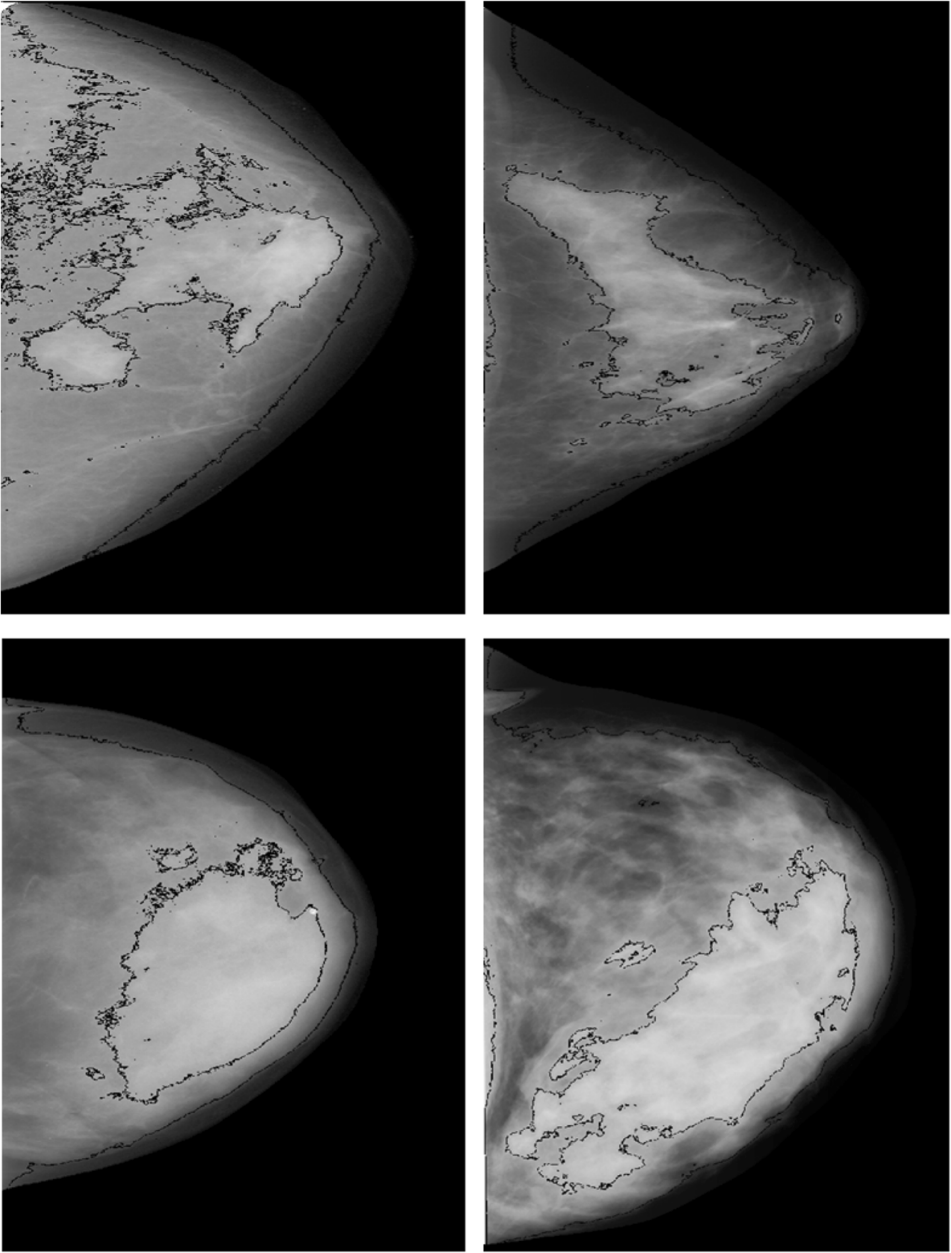
Representative images with similar densities but different groups: *FD_TH_75 values* in the top and bottom 20% of values matched by BMI, PD, age, case status, and study. The top row has similar low PD densities (17%) while the bottom row has a relatively high PD (67%). The inner black delineation lines in each breast image show the delineation lines of the tissue used to describe *FD_TH_75*. The outer black delineation lines show the delineation lines of the tissue used to describe *FD_TH_15*. The top left and bottom left images show a top 20th percent tile value of *FD_TH_75* while the top right and bottom right images show a bottom 20th percent tile value

Figure 2 shows representative images with similar densities but different feature values for the *FD_TH_75* feature. We selected images with *FD_TH_75* values in the top and bottom 20% of values matched by BMI, PD, age, case status, and study. The top row of Fig. 2 has similar low PD densities (17%) while the bottom row has a relatively high PD (67%). The inner black delineation lines in each breast image show the delineation lines of the tissue used to describe *FD_TH_75*. The outer black delineation lines show the delineation lines of the tissue used to describe *FD_TH_15*. The top left and bottom left images show a top 20th percent tile value of FD_TH_75 while the top right and bottom right images show a bottom 20th percent tile value.

In Table 5, the breast cancer risk associated with DCIS and invasive cancer is shown for the 15 most significant features found overall, adjusted for age, BMI, and PD. While invasive cancers have approximately the same significant features as the all-cancer results in Table 2, DCIS showed a smaller number of significant associations with features. *FD_TH_10* and *FD_TH_15* significantly associated with DCIS risk, but not with invasive cancer. Five features were significantly associated with the ER+ cases (Table 5) while no features were significantly associated with ER– status, although power was limited. The patterns of association were similar for risk of DCIS, invasive breast cancer, and ER+ and ER– breast cancer.

**Table 5:** Risk associated of either DCIS or invasive cancer for each feature

Feature	DCIS	Invasive			ER–	ER+		
	OR (95% CI)	OR (95% CI)	*p* value*	*p* het**	OR (95% CI)	OR (95% CI)	*p* value*	*p* het**
*N* case/N control	254/1659	908/1659			116/1291	746/1291		
FD_TH_75	0.87 (0.74–1.01)	0.87 (0.78–0.96)	0.010	0.98	0.84 (0.67–1.06)	0.88 (0.79–0.99)	0.048	0.72
Energy	0.88 (0.76–1.02)	0.88 (0.80–0.96)	0.011	0.93	0.85 (0.69–1.05)	0.86 (0.78–0.95)	0.009	0.90
Entropy	1.18 (1.02–1.38)	1.13 (1.03–1.25)	0.010	0.60	1.16 (0.93–1.44)	1.15 (1.03–1.28)	0.024	0.96
FD_TH_70	0.85 (0.72–1.00)	0.87 (0.79–0.97)	0.015	0.75	0.84 (0.67–1.06)	0.89 (0.79–1.00)	0.085	0.64
FD_TH_80	0.90 (0.77–1.04)	0.89 (0.81–0.98)	0.034	0.90	0.85 (0.68–1.05)	0.89 (0.80–1.00)	0.066	0.64
FD_TH_10	1.19 (1.04–1.38)	1.09 (0.99–1.19)	0.022	0.21	1.03 (0.84–1.26)	1.06 (0.96–1.18)	0.479	0.75
Kurtosis	0.86 (0.73–1.00)	0.90 (0.81–0.99)	0.032	0.58	0.98 (0.78–1.22)	0.91 (0.81–1.01)	0.216	0.53
FD_TH_65	0.84 (0.71–0.99)	0.89 (0.80–0.99)	0.035	0.49	0.83 (0.65–1.06)	0.91 (0.81–1.03)	0.170	0.46
FD_Minkowski	0.90 (0.74–1.08)	0.86 (0.77–0.97)	0.042	0.71	0.77 (0.59–1.01)	0.89 (0.78–1.01)	0.063	0.32
Busyness	1.15 (1.00 –1.33)	1.09 (1.00–1.19)	0.053	0.46	0.92 (0.75–1.14)	1.09 (0.99–1.21)	0.128	0.12
Homogeneity	1.05 (0.90–1.22)	1.13 (1.03–1.24)	0.042	0.36	1.06 (0.86–1.31)	1.12 (1.01–1.24)	0.091	0.62
Dissimilarity	0.96 (0.83–1.12)	0.89 (0.81–0.98)	0.057	0.35	0.95 (0.77–1.17)	0.89 (0.81–0.99)	0.110	0.61
FD_TH_60	0.85 (0.71–1.02)	0.9 (0.8–1.01)	0.077	0.56	0.9 (0.69–1.15)	0.92 (0.81–1.04)	0.348	0.85
FD_TH_85	0.92 (0.79–1.06)	0.91 (0.83–1)	0.130	0.98	0.89 (0.72–1.09)	0.91 (0.82–1.01)	0.173	0.77
FD_TH_15	1.2 (1.04–1.39)	1.06 (0.97–1.16)	0.034	0.09	0.96 (0.78–1.18)	1.05 (0.95–1.16)	0.572	0.43

Results are presented as odds ratio (OR) and 95% confidence interval (CI) per 1 standard deviation in normalized feature after adjustment for age, family history, percent density, and study

*p value refers to two degrees of freedom to test for evidence of association with ductal carcinoma in situ (DCIS) or invasive cancer

**Heterogeneity *p* value (*p* het) to test for differences in effect between tumor subgroups

*ER* estrogen receptor

## Discussion

The combined results of five separate studies, including 1171 cancer cases and 1659 controls, were used to study the association of mammographic textural features on film-screen mammograms, independent of PD, with breast cancer risk overall and defined by tumor type and ER status. Of the 46 features studied, several candidate features demonstrated an association with breast cancer overall. The addition of individual texture features to the baseline model (adjusted for age, BMI, family, PD, and study) demonstrated modest increases in the discriminatory ability of the model. The patterns of association were found to be similar for the risk of DCIS, invasive breast cancer, and ER+ and ER– breast cancer, although there were differences in magnitude of the associations between invasive/DCIS, ER+/ER– status cancer subtypes, and specific features. We also found that many mammographic features associated with breast cancer were not correlated with PD, a desirable quality for potentially improving the discrimination of risk-prediction models. Specifically, the GLCM *Entropy*/*Energy* and *Homogeneity*/*Dissimilarity*, *Busyness*, *FD_15*, and *FD_10* features may be tested in combination with PD in risk-prediction models.

In previous reports, there have been few examples of texture features that are associated with cancer independent of PD. Torres-Mejia et al. [6] found no significant breast cancer risk association of fractal features after adjusting for PD, and Manduca et al. [8] found that features did not add additional significance when adjusted for PD. We found several fractal dimension features associated with breast cancer risk (*FD_TH_5*:*FD_TH_85)*, but the association was reversed dependent on the threshold level used to create the line profiles. An example was given of the *FD_TH_75* (line profile outlining highly dense tissue) and *FD_TH_15* (line profile outlining the edge of the compressed area) in Fig. 1. Thus, the reversal in association from high to low risk is associated with defining fractal characteristics in different types of tissue. Another fractal dimension feature, *FD_Minkowski*, showed a decreased association with cancer risk similar to *FD_TH_75.* These measures are closely mathematically related as noted by their clustering in the dendrogram. Unlike other studies, the association of *FD_Minkowski* feature with breast cancer risk [6] remained significant after adjustment for PD and other risk factors.

Other associated features include the paired features *Entropy* and *Energy* as well as *Homogeneity* and *Dissimilarity*. The *Entropy* is intuitively assumed to be significant for breast cancer risk because tissue with high entropy is more heterogeneous. *Energy* value is associated with a reduced risk of breast cancer because it is related to tissue with more homogeneous texture. The features that denoted more coarseness increased risk and those that were less coarse did not increase risk or were protective. The Pearson correlation coefficients show the features in both pairs are highly negatively correlated. The protective character of *Dissimilarity* (or *Contrast*) is not intuitive. We can speculate that finer structure has high contrast and has similar behavior to fractal dimension. Other studies provided an important role for mammographic textures such as fractal dimensions, GLCM matrix parameters, and power Fourier spectrum in distinguishing between BRCA1/BRCA2 gene mutations and cancer risks [29, 33]. These results are consistent with the results of our study. The fractal dimension and GLCM features derived in our study also demonstrate a significant association with breast cancer risk. The cause and underlying biology of mammographic feature association to breast cancer risk is complex. The features responsible for increased cancer risk are likely to be a measure of image heterogeneity or a degree of local tissue disorganization. Mammograms visualize breast tissue patterns consisting of epithelial and stromal cells, collagen, and fat. These tissue components communicate and interact with each other. Each component may influence the risk and progression of breast cancer [34]. *Entropy* associated with an increased risk of breast cancer and represented a measure of spatial disorder likely to show a degree of tissue heterogeneity. It could be associated with processes on the cellular level where increased entropy is stated to be as a metaphor of progressive irreversible loss of initial order (e.g., by acquiring mutations) in the cell [35]. Another significant feature, *FD_TH_75*, associated with a decreased risk of breast cancer is also related to tissue heterogeneity but in the opposite direction. As shown in Fig. 2 (top right and bottom right images), *FD_TH_75* in the bottom 20th percent tile values represents highly heterogeneous tissue.

Our study had the following limitations. First, many films, especially from the SFMR, were excluded due to temporal inconsistencies with the digitization of cases and controls. Harmonization procedures were needed to rescale the spatial dimensions and dynamic range. Ideally, all images would have been digitized on one digitizer, or been a native digital format (versus film). We also had few ER– and DCIS cancer subtypes, limiting our power for these subtypes. For example, the *FD_TH_10* and *FD_TH_15* features look promising to differentiate DCIS from invasive cancer because, even with fewer cases, they showed significance for DCIS and were not significant for invasive cancers. However, the heterogeneity *p* values to test for differences in effect between DCIS and invasive cancer subgroups were *p* = 0.09 and *p* = 0.21 for *FD_TH_15* and *FD_TH_10,* respectively. Finally, film mammography has largely been replaced by full-field digital mammography systems as well as three-dimensional tomosynthesis systems. However, texture features measured using film mammograms have been shown to be in a good agreement with those measures using digital mammography systems [36]. It is an important point for future validation of the proposed texture features to add MLO view mammograms, to estimate rotation-invariant measures by averaging GLCM features over the four rotations (0, 45, 90, 135 degrees), and to apply them for tomosynthesis slices and projections.

## Conclusions

We conclude that the description of breast density texture from mammograms shows promise as an independent risk factor for breast cancer risk and potentially differentiating between risks of cancer subtypes. For future work, we plan to assess risk prediction combining mammographic density and features assessed on digital mammography and tomosynthesis images.

## Additional files

Additional file 1: Table S1. Baseline characteristics of study population per study site. Table S2. Pearson correlation coefficient for top 15 significant features. Correlations calculated using case subjects. Gray and gray with line pattern highlight strength of positive and negative associations, respectively. (PDF 123 kb)
Additional file 2: Figure S1. Dendrogram of cluster analysis of the top 15 features with PD, age and BMI. Similar features cluster together. Percent density groups closely with BMI and age. The figure is restricted to the cases. (PDF 76 kb)

## Abbreviations

AUC: Area under the curve
BI-RADS: Breast Imaging-Reporting and Data System
BMI: Body mass index
cc: Craniocaudal
CI: Confidence interval
DCIS: Ductal carcinoma in situ
ER: Estrogen receptor
FD: fractal dimension
FT: Fourier transform
GLCM: Gray-level co-occurrence matrix
MCMAM: Mayo Clinic Mammography Study
MMHS: Mayo Mammography Health Study
BGTDM: Neighborhood gray-tone difference matrix
NHS: Nurses’ Health Study
OR: Odds ratio
PD: Percent density
SD: Standard deviation
SFMR: San Francisco Bay Area Breast Cancer SPORE and San Francisco Mammography Registry
UCSF: University of California San Francisco

## References

[1] Tice JA, Cummings SR, Smith-Bindman R, Ichikawa L, Barlow WE, Kerlikowske K (2008). Using clinical factors and mammographic breast density to estimate breast cancer risk: development and validation of a new predictive model. Ann Intern Med.

[2] Boyd NF, Guo H, Martin LJ, Sun L, Stone J, Fishell E, Jong RA, Hislop G, Chiarelli A, Minkin S (2007). Mammographic density and the risk and detection of breast cancer. N Engl J Med.

[3] Wolfe JN (1976). Risk for breast cancer development determined by mammographic parenchymal pattern. Cancer..

[4] ACR (2003). Illustrated breast imaging reporting and data system (BI-RADS).

[5] Wang J, Azziz A, Fan B, Malkov S, Klifa C, Newitt D, Yitta S, Hylton N, Kerlikowske K, Shepherd JA (2013). Agreement of mammographic measures of volumetric breast density to MRI. PLoS One.

[6] Torres-Mejia G, De Stavola B, Allen DS, Perez-Gavilan JJ, Ferreira JM, Fentiman IS, Dos Santos SI (2005). Mammographic features and subsequent risk of breast cancer: a comparison of qualitative and quantitative evaluations in the Guernsey prospective studies. Cancer Epidemiol Biomarkers Prev.

[7] Byng JW, Yaffe M, Lockwood GA, Little LE, Tritchler DL, Boyd NF (1997). Automated analysis of mammographic densities and breast carcinoma risk. Cancer.

[8] Manduca A, Carston MJ, Heine JJ, Scott CG, Pankratz VS, Brandt KR, Sellers TA, Vachon CM, Cerhan JR (2009). Texture features from mammographic images and risk of breast cancer. Cancer Epidemiol Biomark Prev.

[9] Häberle L, Wagner F, Fasching PA, Jud SM, Heusinger K, Loehberg CR, Hein A, Bayer CM, Hack CC, Lux MP (2012). Characterizing mammographic images by using generic texture features. Breast Cancer Res.

[10] Wei J, Chan H-P, Wu Y-T, Zhou C, Helvie MA, Tsodikov A, Hadjiiski LM, Sahiner B (2011). Association of computerized mammographic parenchymal pattern measure with breast cancer risk: a pilot case-control study. Radiology.

[11] Zheng Y, Keller BM, Ray S, Wang Y, Conant EF, Gee JC, Kontos D (2015). Parenchymal texture analysis in digital mammography: a fully automated pipeline for breast cancer risk assessment. Med Phys.

[12] Huo Z, Giger ML, Olopade OI, Wolverton DE, Weber BL, Metz CE, Zhong W, Cummings SA (2002). Computerized analysis of digitized mammograms of BRCA1 and BRCA2 gene mutation carriers 1. Radiology.

[13] Gierach GL, Li H, Loud JT, Greene MH, Chow CK, Lan L, Prindiville SA, Eng-Wong J, Soballe PW, Giambartolomei C (2014). Relationships between computer-extracted mammographic texture pattern features and BRCA1/2 mutation status: a cross-sectional study. Breast Cancer Res.

[14] Li H, Giger ML, Sun C, Ponsukcharoen U, Huo D, Lan L, Olopade OI, Jamieson AR, Brown JB, Di Rienzo A (2014). Pilot study demonstrating potential association between breast cancer image-based risk phenotypes and genomic biomarkers. Med Phys.

[15] Keller BM, Chen J, Conant EF, Kontos D. Breast density and parenchymal texture measures as potential risk factors for estrogen-receptor positive breast cancer. In SPIE Medical Imaging. Bellingham: International Society for Optics and Photonics; 2014. pp. 90351D–90351D. 10.1117/12.2043710 PMC4112103 25075270

[16] Bertrand KA, Tamimi RM, Scott CG, Jensen MR, Pankratz VS, Visscher D, Norman A, Couch F, Shepherd J, Fan B (2013). Mammographic density and risk of breast cancer by age and tumor characteristics. Breast Cancer Res.

[17] Bertrand KA, Scott CG, Tamimi RM, Jensen MR, Pankratz VS, Norman AD, Visscher DW, Couch FJ, Shepherd J, Chen Y-Y. Dense and nondense mammographic area and risk of breast cancer by age and tumor characteristics. Cancer Epidemiol Biomarkers Prev. 2015;24(5):798–809. 10.1158/1055-9965.EPI-14-1136 PMC4417380 25716949

[18] Olson JE, Sellers TA, Scott CG, Schueler BA, Brandt KR, Serie DJ, Jensen MR, Wu F-F, Morton MJ, Heine JJ (2012). The influence of mammogram acquisition on the mammographic density and breast cancer association in the Mayo mammography health study cohort. Breast Cancer Res.

[19] Colditz GA (2005). Estrogen, estrogen plus progestin therapy, and risk of breast cancer. Clin Cancer Res.

[20] Vachon CM, van Gils CH, Sellers TA, Ghosh K, Pruthi S, Brandt KR, Pankratz VS (2007). Mammographic density, breast cancer risk and risk prediction. Breast Cancer Res.

[21] Kerlikowske K, Shepherd J, Creasman J, Tice JA, Ziv E, Cummings SR (2005). Are breast density and bone mineral density independent risk factors for breast cancer?. J Natl Cancer Inst.

[22] Byng J, Boyd N, Fishell E, Jong R, Yaffe M (1996). Automated analysis of mammographic densities. Phys Med Biol.

[23] Shepherd JA, Kerlikowske K, Ma L, Duewer F, Fan B, Wang J, Malkov S, Vittinghoff E, Cummings SR (2011). Volume of mammographic density and risk of breast cancer. Cancer Epidemiol Biomarkers Prev.

[24] Castella C, Kinkel K, Eckstein MP, Sottas P-E, Verdun FR, Bochud FO (2007). Semiautomatic mammographic parenchymal patterns classification using multiple statistical features. Acad Radiol.

[25] Mavroforakis ME, Georgiou HV, Dimitropoulos N, Cavouras D, Theodoridis S (2006). Mammographic masses characterization based on localized texture and dataset fractal analysis using linear, neural and support vector machine classifiers. Artif Intell Med.

[26] Burgess AE. Mammographic structure: Data preparation and spatial statistics analysis. In Medical Imaging'99. Bellingham: International Society for Optics and Photonics; 1999. pp. 642–653.

[27] Haralick RM, Shanmugam K, Dinstein IH (1973). Textural features for image classification. Syst Man Cybernetics IEEE Trans..

[28] Amadasun M, King R (1989). Textural features corresponding to textural properties. IEEE Trans Syst Man Cybern.

[29] Li H, Giger ML, Olopade OI, Margolis A, Lan L, Chinander MR (2005). computerized texture analysis of mammographic parenchymal patterns of digitized mammograms 1. Acad Radiol.

[30] Caldwell CB, Stapleton SJ, Holdsworth DW, Jong RA, Weiser WJ, Cooke G, Yaffe MJ (1990). Characterisation of mammographic parenchymal pattern by fractal dimension. Phys Med Biol.

[31] Boone JM, Lindfors KK, Beatty CS, Seibert JA (1998). A breast density index for digital mammograms based on radiologists’ ranking. J Digit Imaging.

[32] Malkov S, Mahmoudzadeh AP, Kerlikowske K, Shepherd J. Automated Volumetric Breast Density Derived by Statistical Model Approach. In International Workshop on Digital Mammography. Cham: Springer International Publishing; 2014. pp. 257–264.

[33] Li H, Giger ML, Olopade OI, Lan L (2007). Fractal analysis of mammographic parenchymal patterns in breast cancer risk assessment. Acad Radiol.

[34] Boyd NF, Martin LJ, Bronskill M, Yaffe MJ, Duric N, Minkin S. Breast tissue composition and susceptibility to breast cancer. J Nat Cancer Inst. 2010;102(16):1. 10.1093/jnci/djq239 PMC2923218 20616353

[35] Tarabichi M, Antoniou A, Saiselet M, Pita JM, Andry G, Dumont JE, Detours V, Maenhaut C (2013). Systems biology of cancer: entropy, disorder, and selection-driven evolution to independence, invasion and “swarm intelligence”. Cancer Metastasis Rev.

[36] Jing H, Yang YY, Wernick MN, Yarusso LM, Nishikawa RM (2012). A comparison study of image features between FFDM and film mammogram images. Med Phys.

