# Erratum to: Mammographic texture and risk of breast cancer by tumor type and estrogen receptor status

**DOI:** 10.1186/s13058-016-0797-y

**Published:** 2017-01-04

**Authors:** Serghei Malkov, John A. Shepherd, Christopher G. Scott, Rulla M. Tamimi, Lin Ma, Kimberly A. Bertrand, Fergus Couch, Matthew R. Jensen, Amir P. Mahmoudzadeh, Bo Fan, Aaron Norman, Kathleen R. Brandt, V. Shane Pankratz, Celine M. Vachon, Karla Kerlikowske

**Affiliations:** 1 Department of Radiology and Biomedical Imaging, UCSF School of Medicine, San Francisco, CA USA; 2 Mayo Clinic, Rochester, MN USA; 3 UCSF Departments of Medicine and Epidemiology/Biostatistics, San Francisco, CA USA; 4 Harvard Medical School, Boston, MA USA; 5 Slone Epidemiology Center at Boston University, Boston, MA USA

## Erratum

After publication of the original article [1], the author noticed some errors to the article [1] which are included in this erratum. All errors reported in this erratum have been updated in the original article [1].

In the ‘Results’ section of the article Abstract, the sentence “Entropy was associated with a decreased risk of breast cancer” should be "Entropy was associated with an increased risk of breast cancer”. In the same section, the variables "*FD_TH10*", "*FD_TH15*", "*FD_TH60*", "*FD_TH85*", "*FD_TH75*", and "FD_TH75" should include an additional underscore so they are presented as: "*FD_TH_10*", "*FD_TH_15*", "*FD_TH_60*", "*FD_TH_85*", "*FD_TH_75*", and "*FD_TH_75*", respectively. All variables should be italicized.

In the section entitled ‘Breast texture measurements’, the sentence “the image with only constant grayscale pixels has *Energy* equal to 0" should state "the image with only constant grayscale pixels has *Energy* equal to 1".

In the ‘Results’ section of the article [1], the variable “FD_TH_75” included in sentence: “The top left and bottom left images show a top 20th percent tile value of FD_TH_75” should be included in italics.

In the ‘Abbreviations’ section, “BGTDM” should be "NGTDM". The correct version of the ‘Abbreviations’ is included in this erratum and has been updated in the original article [1].

In Table 1, in the column “Texture feature name”, the names of variables should be in italics. In addition in Table 5, in the column “Feature”, all variables should also be included in italics. The revised versions of Tables 1 and 5 are included in this erratum and have been updated in the original article [1].

**Table 1:** Image texture features that are currently defined for all study participants

Analysis Groups	Texture Features	Texture feature name	Reference
Gray-level Histogram	Standard deviation	*STD*	[7, 22, 24–26]
	Skewness	*Skewness*	
	Kurtosis	*Kurtosis*	
	Balance	*Balance*	
Grey-level co-occurrence matrix (GLCM)	GLCM Energy	*Energy*	[24, 25, 27, 29]
	GLCM Entropy	*Entropy*	
	GLCM Dissimilarity	*Dissimilarity*	
	GLCM Contrast	*Contrast*	
	GLCM Homogeneity	*Homogeneity*	
	GLCM Correlation	*Correlation*	
	GLCM Mean	*GLCM Mean*	
	GLCM Variance	*GLCM Variance*	
Neighborhood Gray-tone Difference Matrix (NGTDM)	NGTDM Coarseness	*NGTDM Coarseness*	[24, 28, 29]
	NGTDM Contrast	*NGTDM Contrast*	
	NGTDM Complexity	*Complexity*	
	NGTDM Strength	*Strength*	
	NGTDM Busyness	*Busyness*	
Edge Frequency Analysis	Mean Gradient	*Mean_Gradient*	[29]
Fourier Transform Analysis, Power Spectrum	RMS	*FT_RMS*	[29]
	FMP (first moment of power spectrum)	*FT_FMP*	
	SMP (second moment of power spectrum)	*FT_SMP*	
	FD from power spectrum exponent	*FT_FD*	
Fractal Analysis	Intercept of the plot of the standard deviation of the high frequency image as a function of the size the kernel	*CD_Yint*	[29–31]
	Continuous Dimension (CD), slope and intercept	*CD_Slope*	
	HZ_PROJ	*HZ_PROJ*	
	FD of the standard deviation	*FD_Sigma*	
	FD of image using thresholds from 5%-85%	*FD_TH_5: FD_TH_85*	
	FD of the surface of thebreast considering the gray value represent the height	*FD_CALDWELL*	
	FD, Minkowski method	*FD_Minkowski*

**Table 5:** Risk associated of either DCIS or Invasive Cancer for each feature

Feature	DCIS	Invasive			ER-	ER+		
	OR (95% CI)	OR (95% CI)	p-value*	p-het**	OR (95% CI)	OR (95% CI)	*p*-value *	p-het**
N case/control	*254/1659*	*908/1659*			*116/1291*	*746/1291*		
*FD_TH_75*	0.87 (0.74, 1.01)	0.87 (0.78, 0.96)	0.010	0.98	0.84 (0.67, 1.06)	0.88 (0.79, 0.99)	0.048	0.72
*Energy*	0.88 (0.76, 1.02)	0.88 (0.80, 0.96)	0.011	0.93	0.85 (0.69, 1.05)	0.86 (0.78, 0.95)	0.009	0.90
*Entropy*	1.18 (1.02, 1.38)	1.13 (1.03, 1.25)	0.010	0.60	1.16 (0.93, 1.44)	1.15 (1.03, 1.28)	0.024	0.96
*FD_TH_70*	0.85 (0.72, 1.00)	0.87 (0.79, 0.97)	0.015	0.75	0.84 (0.67, 1.06)	0.89 (0.79, 1.00)	0.085	0.64
*FD_TH_80*	0.90 (0.77, 1.04)	0.89 (0.81, 0.98)	0.034	0.90	0.85 (0.68, 1.05)	0.89 (0.80, 1.00)	0.066	0.64
*FD_TH_10*	1.19 (1.04, 1.38)	1.09 (0.99, 1.19)	0.022	0.21	1.03 (0.84, 1.26)	1.06 (0.96, 1.18)	0.479	0.75
*Kurtosis*	0.86 (0.73, 1.00)	0.90 (0.81, 0.99)	0.032	0.58	0.98 (0.78, 1.22)	0.91 (0.81, 1.01)	0.216	0.53
*FD_TH_65*	0.84 (0.71, 0.99)	0.89 (0.80, 0.99)	0.035	0.49	0.83 (0.65, 1.06)	0.91 (0.81, 1.03)	0.170	0.46
*FD_Minkowski*	0.90 (0.74, 1.08)	0.86 (0.77, 0.97)	0.042	0.71	0.77 (0.59, 1.01)	0.89 (0.78, 1.01)	0.063	0.32
*Busyness*	1.15 (1.00, 1.33)	1.09 (1.00, 1.19)	0.053	0.46	0.92 (0.75, 1.14)	1.09 (0.99, 1.21)	0.128	0.12
*Homogeneity*	1.05 (0.90, 1.22)	1.13 (1.03, 1.24)	0.042	0.36	1.06 (0.86, 1.31)	1.12 (1.01, 1.24)	0.091	0.62
*Dissimilarity*	0.96 (0.83, 1.12)	0.89 (0.81, 0.98)	0.057	0.35	0.95 (0.77, 1.17)	0.89 (0.81, 0.99)	0.110	0.61
*FD_TH_60*	0.85 (0.71, 1.02)	0.9 (0.8, 1.01)	0.077	0.56	0.9 (0.69, 1.15)	0.92 (0.81, 1.04)	0.348	0.85
*FD_TH_85*	0.92 (0.79, 1.06)	0.91 (0.83, 1)	0.130	0.98	0.89 (0.72, 1.09)	0.91 (0.82, 1.01)	0.173	0.77
*FD_TH_15*	1.2 (1.04, 1.39)	1.06 (0.97, 1.16)	0.034	0.09	0.96 (0.78, 1.18)	1.05 (0.95, 1.16)	0.572	0.43

Results presented as OR per 1 SD in normalized feature after adjustment for age, family history, PD, and study

*p-value refers to 2 degree of freedom to test for evidence of associated with DCIS or invasive cancer

**Heterogeneity p-value to test for differences in effect between tumor subgroups

## Abbreviations

AUC: Area under the curve
BI-RADS: Breast Imaging-Reporting and Data System
BMI: Body mass index
cc: Craniocaudal
CI: Confidence interval
DCIS: Ductal carcinoma in situ
ER: Estrogen receptor
FD: fractal dimension
FT: Fourier transform
GLCM: Gray-level co-occurrence matrix
MCMAM: Mayo Clinic Mammography Study
MMHS: Mayo Mammography Health Study
NGTDM: Neighborhood gray-tone difference matrix
NHS: Nurses’ Health Study
OR: Odds ratio
PD: Percent density
SD: Standard deviation
SFMR: San Francisco Bay Area Breast Cancer SPORE and San Francisco Mammography Registry
UCSF: University of California San Francisco
