# Supplementary material for: Mammographic texture and risk of breast cancer by tumor type and estrogen receptor status
Source: Breast Cancer Res. 2016 Dec 6;18:122. doi: 10.1186/s13058-016-0778-1 (PMC5139106; doi:10.1186/s13058-016-0778-1)
Supplement: Additional file 1: Table S1. — Baseline characteristics of study population per study site. Table S2. Pearson correlation coefficient for top 15 significant features. Correlations calculated using case subjects. Gray and gray with line pattern highlight strength of positive and negative associations, respectively. (PDF 123 kb) [file 13058_2016_778_MOESM1_ESM.pdf]

Table S1. Baseline characteristics of study population by site**

	MMHS		NHS		NHSII		MCMAM		SFMR	
	Cases	Controls	Cases	Controls	Cases	Controls	Cases	Controls	Cases	Controls
**N**	62	112	412	454	351	492	242	395	104	206
**Mean age at mammogram (SD)**	55.8 (9.3)	53.3 (7.2)	58.6 (7.7)	58.3 (7.7)	45.5 (4.3)	45.9 (4.3)	64.1 (10.1)	64.1 (10.1)	55.5 (11.3)	55.5 (11.3)
**Mean age at diagnosis (SD)**	58.3 (9.3)	--	66 (6.9)	--	49.7 (4.3)	--	67.8 (10.1)	--	59.8 (11.3)	--
**Mean BMI (SD)**	29.7 (6.7)	29.9 (7.9)	26.1 (4.9)	25.8 (5.4)	24.3 (5.8)	25.2 (7.7)	26.6 (7.6)	26.8 (6.8)	24.2 (5.6)	23.5 (4.7)
**Body mass index categories, kg/m^2**										
<25	16 (26%)	36 (32%)	186 (45%)	230 (51%)	**206 (59%)**	**270 (54.9%)**	80 (33%)	163 (41%)	63 (61%)	155 (75%)
25-29	17 (27%)	27 (24%)	139 (34%)	136 (30%)	**99 (28%)**	**111 (22.6%)**	86 (35%)	128 (32%)	28 (27%)	41 (20%)
30-34	16 (26%)	25 (22%)	63 (15%)	55 (12%)	**25 (7%)**	**47 (9.6%)**	44 (18%)	62 (16%)	7 (7%)	7 (3%)
35+	13 (21%)	24 (21%)	22 (5%)	29 (6%)	**13 (4%)**	**48 (9.8%)**	21 (9%)	32 (8%)	4 (4%)	3 (2%)
Unknown	0 (0%)	0 (0%)	2 (1%)	4 (1%)	**8 (2%)**	**16 (3.3%)**	11 (5%)	10 (3%)	2 (2%)	0 (0%)
**Menopausal Status**										
Premenopausal	18 (29%)	42 (37%)	75 (18%)	99 (22%)	266 (76%)	361 (73.4%)	38 (16%)	67 (17%)	33 (32%)	63 (31%)
Postmenopausal	44 (71%)	69 (62%)	336 (82%)	355 (78%)	55 (16%)	89 (18.1%)	202 (83%)	324 (82%)	60 (58%)	125 (61%)
Unknown	0 (0%)	1 (1%)	1 (0.2%)	0 (0%)	30 (8%)	42 (8.5%)	2 (1%)	4 (1%)	11 (11%)	18 (9%)
**Parity**										
Nulliparous	5 (8%)	18 (16%)	**34 (8%)**	**15 (3%)**	64 (18%)	86 (17.5%)	33 (14%)	48 (12%)	33 (32%)	51 (25%)
Parous	57 (92%)	87 (78%)	**371 (90%)**	**435 (96%)**	285 (81%)	405 (82.3%)	209 (86%)	346 (88%)	55 (53%)	135 (65%)
Unknown	0 (0%)	7 (6%)	**7 (2%)**	**4 (1%)**	2 (1%)	1 (0.2%)	0 (0%)	1 (0.3%)	16 (15%)	20 (10%)
**Postmenopausal hormone therapy***										
Not current	38 (86%)	52 (75%)	161 (48%)	192 (54%)	**11 (20%)**	**28 (31.5%)**	--	--	45 (75%)	95 (76%)
Current, estrogen	3 (7%)	8 (12%)	91 (27%)	95 (27%)	**18 (33%)**	**45 (50.6%)**	--	--	4 (7%)	8 (6%)
Current, estrogen + progestin	3 (7%)	9 (13%)	84 (25%)	68 (19%)	**26 (47%)**	**16 (18%)**	--	--	11 (18%)	22 (18%)
**Family History**										
No	44 (71%)	90 (80%)	347 (84%)	400 (88%)	**298 (85%)**	**443 (90%)**	200 (83%)	338 (86%)	84 (81%)	182 (88%)
Yes	18 (29%)	22 (20%)	65 (16%)	54 (12%)	**53 (15%)**	**49 (10%)**	42 (17%)	57 (14%)	18 (17%)	24 (12%)
Unknown	0 (0%)	0 (0%)	0 (0%)	0 (0%)	**0 (0%)**	**0 (0%)**	0 (0%)	0 (0%)	2 (2%)	0 (0%)
**Standardized Mean % mammographic density (SD)**	32.5 (19.0)	28.8 (17.9)	**29.1 (16)**	**24.8 (15.4)**	**39.6 (19.3)**	**33.6 (20.2)**	**30 (18.2)**	**24.5 (17.9)**	**32.3 (21.2)**	**26.7 (17.6)**
**Standardized Mean Dense Area cm^2 (SD)**	54.7 (38.2)	45.8 (28.2)	**71.4 (45.5)**	**60.8 (38.6)**	**58.5 (34.2)**	**48.3 (32.6)**	**51.8 (36.2)**	**42.2 (32.4)**	**80.9 (62.3)**	**65.2 (51.5)**
**Standardized Mean Nondense Area cm^2 (SD)**	149.6 (113.1)	152.1 (122.0)	**187.8 (87.1)**	**207.5 (96.0)**	**98.7 (63.0)**	**111.6 (73.3)**	138.3 (91.3)	145.3 (85.7)	194.4 (151.0)	194.6 (129.1)

Data presentation: mean (standard deviation) or number (%).
*Among postmenopausal women in MMHS, NHS, NHSII , and SFMR
**Sites generally matched cases and controls on age and date of mammogram
Bold denotes statistically significant difference between cases and controls within study (p-value < 0.05)

Table S2. Pearson correlation coefficient for top 15 significant features. Correlations calculated using case subjects. Gray and gray with line pattern highlight strength of positive and negative associations respectively.

	Homogeneity	*Energy*	*FD_TH_75*	*FD_TH_70*	*FD_TH_80*	*FD_TH_65*	*FD_TH_85*	*FD_TH_60*	*Kurtosis*	*Minkowski FD*	*FD_TH_15*	*FD_TH_10*	*Busyness*	PD	*Entropy*	*Dissimilarity*
**Homogeneity**	1	0.37	0.02	-0.01	0.01	-0.01	0.02	-0.01	0.03	-0.26	-0.22	-0.2	-0.04	-0.05	-0.46	-0.98
Energy	0.37	1	0.2	0.18	0.23	0.15	0.25	0.11	0.13	-0.19	-0.17	-0.2	-0.09	-0.01	-0.82	-0.33
FD_TH_75	0.02	0.2	1	0.94	0.95	0.89	0.88	0.82	0.37	0.43	-0.06	-0.11	-0.42	-0.32	-0.41	-0.04
FD_TH_70	-0.01	0.18	0.94	1	0.88	0.96	0.79	0.91	0.42	0.5	-0.04	-0.09	-0.39	-0.39	-0.42	-0.02
FD_TH_80	0.01	0.23	0.95	0.88	1	0.81	0.95	0.73	0.29	0.35	-0.07	-0.11	-0.43	-0.24	-0.4	-0.02
FD_TH_65	-0.01	0.15	0.89	0.96	0.81	1	0.7	0.96	0.48	0.55	-0.03	-0.09	-0.36	-0.46	-0.42	-0.02
FD_TH_85	0.02	0.25	0.88	0.79	0.95	0.7	1	0.62	0.21	0.26	-0.07	-0.11	-0.41	-0.14	-0.37	-0.02
FD_TH_60	-0.01	0.11	0.82	0.91	0.73	0.96	0.62	1	0.52	0.58	-0.03	-0.08	-0.34	-0.52	-0.4	-0.03
Kurtosis	0.03	0.13	0.37	0.42	0.29	0.48	0.21	0.52	1	0.38	-0.3	-0.5	-0.27	-0.34	-0.53	-0.06
FD_Minkowski	-0.26	-0.19	0.43	0.5	0.35	0.55	0.26	0.58	0.38	1	0.44	0.34	0.18	-0.55	-0.11	0.21
FD_TH_15	-0.22	-0.17	-0.06	-0.04	-0.07	-0.03	-0.07	-0.03	-0.3	0.44	1	0.85	0.62	-0.15	0.19	0.25
FD_TH_10	-0.2	-0.2	-0.11	-0.09	-0.11	-0.09	-0.11	-0.08	-0.5	0.34	0.85	1	0.56	-0.1	0.3	0.22
Busyness	-0.04	-0.09	-0.42	-0.39	-0.43	-0.36	-0.41	-0.34	-0.27	0.18	0.62	0.56	1	-0.03	0.2	0.09
**PD**	-0.05	-0.01	-0.32	-0.39	-0.24	-0.46	-0.14	-0.52	-0.34	-0.55	-0.15	-0.1	-0.03	1	0.23	0.09
Entropy	-0.46	-0.82	-0.41	-0.42	-0.4	-0.42	-0.37	-0.4	-0.53	-0.11	0.19	0.3	0.2	0.23	1	0.44
Dissimilarity	-0.98	-0.33	-0.04	-0.02	-0.02	-0.02	-0.02	-0.03	-0.06	0.21	0.25	0.22	0.09	0.09	0.44	1
