# Supplementary material for: Mammographic texture and risk of breast cancer by tumor type and estrogen receptor status
Source: Breast Cancer Res. 2016 Dec 6;18:122. doi: 10.1186/s13058-016-0778-1 (PMC5139106; doi:10.1186/s13058-016-0778-1)
Supplement: Additional file 2: Figure S1. — Dendrogram of cluster analysis of the top 15 features with PD, age and BMI. Similar features cluster together. Percent density groups closely with BMI and age. The figure is restricted to the cases. (PDF 76 kb) [file 13058_2016_778_MOESM2_ESM.pdf]

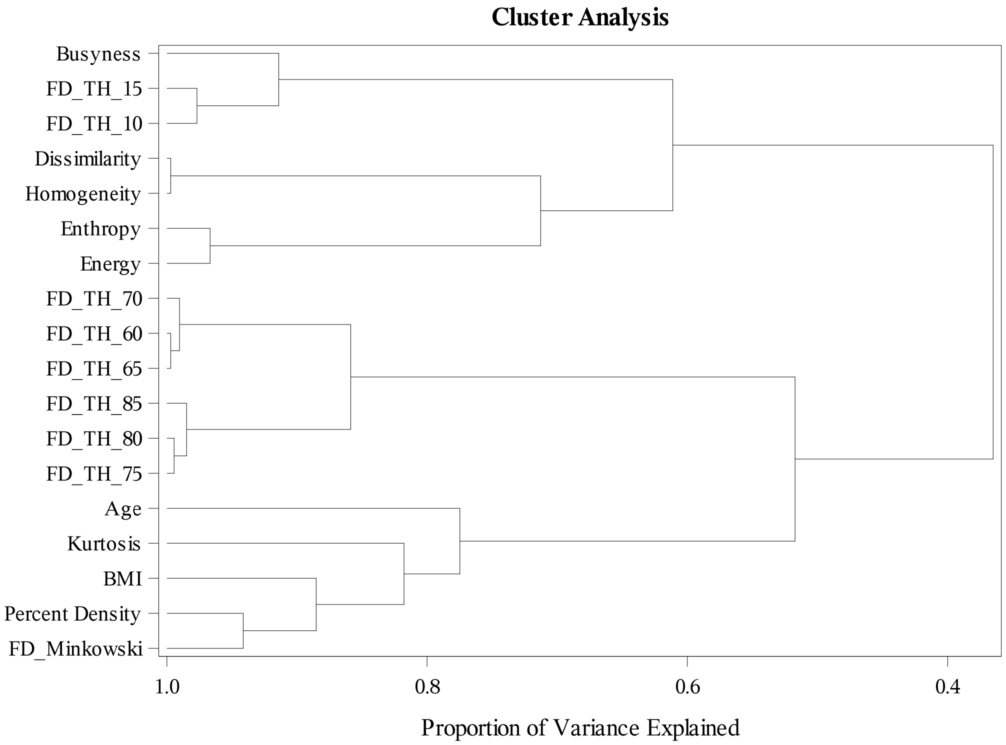

Figure S1. Dendrogram of cluster analysis of the top 15 features with PD, age and BMI. Similar features cluster together. Percent Density groups closely with BMI and age. The figure is restricted to the cases.
